# A Bulk-Heterostructure Nanocomposite Electrolyte of Ce_0.8_Sm_0.2_O_2-δ_–SrTiO_3_ for Low-Temperature Solid Oxide Fuel Cells

**DOI:** 10.1007/s40820-020-00574-3

**Published:** 2021-01-04

**Authors:** Yixiao Cai, Yang Chen, Muhammad Akbar, Bin Jin, Zhengwen Tu, Naveed Mushtaq, Baoyuan Wang, Xiangyang Qu, Chen Xia, Yizhong Huang

**Affiliations:** 1 State Key Laboratory for Modification of Chemical Fibers and Polymer Materials, Key Laboratory of High Performance Fibers and Products, Engineering Research Center of Technical Textiles, Ministry of Education, College of Materials Science and Engineering, Donghua University, 201620 Shanghai, People’s Republic of China; 2 Key Laboratory of Ferro and Piezoelectric Materials and Devices of Hubei Province, Faculty of Physics and Electronic Science, Hubei University, Wuhan, 430062 Hubei People’s Republic of China; 3 School of Materials Science and Engineering, Nanyang Technological University, Singapore, 639798 Republic of Singapore

**Keywords:** Bulk-heterostructure, SOFC electrolyte, Ionic conductivity, Schottky junction, Work function

## Abstract

Since colossal ionic conductivity was detected in the planar heterostructures consisting of fluorite and perovskite, heterostructures have drawn great research interest as potential electrolytes for solid oxide fuel cells (SOFCs). However, so far, the practical uses of such promising material have failed to materialize in SOFCs due to the short circuit risk caused by SrTiO_3_. In this study, a series of fluorite/perovskite heterostructures made of Sm-doped CeO_2_ and SrTiO_3_ (SDC–STO) are developed in a new bulk-heterostructure form and evaluated as electrolytes. The prepared cells exhibit a peak power density of 892 mW cm^−2^ along with open circuit voltage of 1.1 V at 550 °C for the optimal composition of 4SDC–6STO. Further electrical studies reveal a high ionic conductivity of 0.05–0.14 S cm^−1^ at 450–550 °C, which shows remarkable enhancement compared to that of simplex SDC. Via AC impedance analysis, it has been shown that the small grain-boundary and electrode polarization resistances play the major roles in resulting in the superior performance. Furthermore, a Schottky junction effect is proposed by considering the work functions and electronic affinities to interpret the avoidance of short circuit in the SDC–STO cell. Our findings thus indicate a new insight to design electrolytes for low-temperature SOFCs.

**Supplementary Information:**

The online version contains supplementary material available at 10.1007/s40820-020-00574-3.

## Highlights

- Bulk-heterostructure electrolytes based on Ce_0.8_Sm_0.2_O_2-δ_ and SrTiO_3_ are developed for solid oxide fuel cells
- The interface characteristics are investigated to understand the fast ionic transport obtained in the heterostructure.
- The Schottky junction effect is proposed by taking account of work functions and electronic affinities for the first time.

**Supplementary Information:**

The online version contains supplementary material available at 10.1007/s40820-020-00574-3.

## Graphical abstract

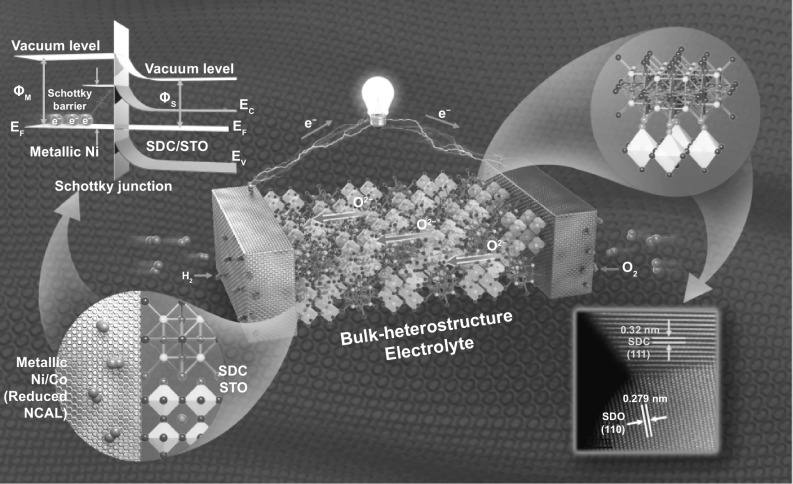

## Introduction

Fast ionic transport is highly desired by solid oxide fuel cells (SOFCs), as high ionic conduction of electrolytes and electrodes is directly linked to superb power outputs, robust durability, and the rapid start-up of fuel cells [1, 2]. However, the electrolyte always requires ionic conductivity as high as 0.1 S cm^−1^ to achieve favorable performance. This leads to strict temperatures of above 800 °C for SOFCs to operate, due to the fact that ionic transport is thermally motivated. Typically, the most frequently used electrolyte, Y_2_O_3_-stabilized ZrO_2_ (YSZ), demands a high temperature of ~1000 °C to attain sufficient oxygen ionic conductivity to run the fuel cell, leading to high costs and technological complexity that could hamper the advance of SOFCs. Therefore, developing low-temperature (LT) electrolytes capable of high ionic conduction at <600 °C has become increasingly accepted as a mainstream in the SOFC community [3–5].

To develop desirable electrolytes, extensive efforts have been dedicated to investigating defect modulation of ceria and lanthanum gallate, reduction of YSZ thickness by thin-film-based techniques, and structural design. Use of these potent strategies has led to modest reductions in the operating temperature of SOFCs down to 500–700 °C [6–8]. Among these, planar heterostructure materials enable high ionic conduction at even lower temperatures. Garcia-Barriocanal et al. reported a colossal ionic conductivity of ~0.1 S cm^−1^ at 200 °C in an ultrathin YSZ/SrTiO_3_ (YSZ/STO) epitaxial heterostructure which exhibited eight orders of magnitude enhancement in comparison with pure YSZ [9]. Since then, a series of studies have been proposed to design artificial oxide planar heterostructures for high oxygen ion conductivity. Yang et al. reported on a heterostructure composed of nanocolumn Sm-doped CeO_2_ (SDC) embedded in STO substrate, which exhibited ionic conductivity increase of more than one order of magnitude as compared to plain SDC films [10]. The remarkable ionic enhancement is ascribed to the highly disordered oxygen plane at the fluorite/perovskite interface, where the combination of massive mobile ions with the opening out of the fluorite lattice led to high interfacial conductivity [11].

However, thereafter the expected practical application of these heterostructure materials failed to materialize even though astonishing ionic conductivity is still being discovered in such system [12, 13]. This is primarily due to the controversial viewpoints on the origin of the conductivity increase and the suspicion of high rate of electron/hole transport in STO that is unfavorable for electrolyte [14]. As a result, the utilization of YSZ/STO and SDC/STO in LT-SOFCs has completely stagnated in recent years. To break this predicament, the negative influence of electrons must be firstly eliminated while retaining the high ionic conduction. Taking some special charge carrier behaviors of semiconductors into account, the electronic transport in these heterostructures can be modulated. For instance, perovskite SmNiO_3_ (SNO) has been utilized as an SOFC electrolyte through a filling-controlled Mott transition to suppress its electrons [15]; the electron–hole pairs in the absorption layer of a thin-film solar cell can be separated by p–n junction [16]; and a sequence of semiconductors have been proposed for electrolyte purpose in a heterostructure composite form to suppress their electronic conduction [17–20]. These works manifest the electronic elimination of YSZ/STO and SDC/STO can be anticipated.

Following the above demonstrations, we further promoted the SDC/STO system from a two-dimensional planar heterostructure to a three-dimensional heterostructure composite for electrolyte uses. A bulk-heterostructure SDC–STO was rationally designed with competent electrolyte functionalities. Electrical studies in terms of polarization curves tests and AC impedance analysis verify the remarkable ionic conductivity of the optimal SDC–STO. The interface properties of the material were studied to understand its conducting behavior. A metal/semiconductor Schottky junction effect was also investigated to describe how the device avoids short circuit risk. This work thus provides an effective strategy to construct fluorite/perovskite heterostructure for SOFC electrolytes.

## Experimental Section

### Material Preparation

The SDC used in this study, Ce_0.8_Sm_0.2_O_2-δ_, was synthesized by a co-precipitation method. Stoichiometric amounts of precursors Ce(NO_3_)_2_·6H_2_O and Sm(NO_3_)_2_·6H_2_O (Sigma-Aldrich, USA) were dissolved in deionized water with continuous stirring to form a 1 M solution. Afterward, 1 M Na_2_CO_3_ (Sigma-Aldrich, USA) solution as the precipitation agent was dropwise added into the above nitrate solution according to a molar ratio of metal ion: carbonate ion=1:1.5 where the precipitation could be produced. Subsequently, the precipitate was repeatedly filtrated and washed, then later dried at 120 °C for 24 h, followed by sintering at 800 °C for 4 h and adequate grinding to obtain the SDC powder.

The bulk-heterostructure SDC–STO powder materials were prepared via a solid-state mixing procedure by ball milling the resultant SDC and a commercial STO powders (Sigma-Aldrich, USA) in various mass ratios (7:3, 6:4, 5:5, 4:6). The ball milling was performed on a planetary ball mill (XQM-0.4 L) operated at 400 rpm for 10 h under atmospheric pressure, during which ethanol was used as the dispersing medium. The used commercial powder is a kind of perovskite nanostructured SrTiO_3_ with particle size of < 100 nm and purity of 99.5%. It is a typical non-fluorite substrate for doped ceria films to be deposited to maintain the correct stoichiometry, as the lattice mismatch between them is small. The mixture powders were then sintered at 700 °C for 2 h and ground completely to obtain SDC–STO samples.

### Fuel Cell Fabrication

SOFCs based on the SDC–STO electrolytes with various ratios were fabricated via a dry pressing method. A semiconductor NCAL was used as a symmetrical electrode in the form of NCAL-pasted Ni-foam (NCAL-Ni), which has been reported recently as a competent catalyst with H^+^/O^2−^/e^−^ triple conduction and good activity for both hydrogen oxidation reaction (HOR) and oxygen reduction reaction (ORR) [19]. The NCAL-Ni electrodes were prepared by blending NCAL powders with terpineol solvent to form slurry, which was then pasted on Ni-foam followed by desiccation at 150 ℃ for 1 h to form NCAL-Ni components. The Ni-foam was used to guarantee the mechanical strength of the cell and sustain the porous structure of the electrode.

Following a typical fuel cell fabrication procedure, the SDC–STO powder was compacted between two pieces of NCAL-Ni electrodes uniaxially under a pressure load of 200 MPa into one pellet. The cell pellets with various proportions of SDC–STO were assembled in a same configuration of *NCAL-Ni/SDC–STO/NCAL-Ni*, with thicknesses of ~1.5 mm and 0.64 cm^2^ in the active area. The thickness of electrolyte is approximately 500 μm. Additionally, for comparative study, a fuel cell with a single SDC electrolyte (*NCAL-Ni/SDC/NCAL-Ni*) was also fabricated using the same procedure and cell size. All these fuel cells were brushed with silver paste onto the electrode surface as a current collector and for gas sealing before being mounted into the testing jig. This was followed by online sintering at 650 °C for 2 h prior to operation and performance measurement.

### Material Characterizations and Electrochemical Measurements

The crystal structures of the SDC, STO, and SDC–STO bulk-heterostructures were analyzed by Bruker D8 Advanced X-ray diffractometer (XRD) with a Cu Kα (λ=1.54060 Å) source, a tube voltage of 45 kV, and a current of 40 mA. The diffraction patterns were recorded in the 2θ range of 20°–80° with intervals of 0.02°. The microstructures of these specimens were investigated using a transmission electron microscope (TEM, JEOL JEM-2100F) operating under an accelerating voltage of 200 kV. Further interface investigations were performed using a JEOL ARM 200 CF microscope equipped with a cold field emission electron source. Collection semi angles of 111.5 and 57.1 mrads were used to record the electron energy-loss spectroscopy (EELS) elemental mappings and line scans. The work functions of the materials were attained via ultraviolet photoelectron spectroscopy (UPS) measurements performed with an unfiltered HeI (21.22 eV) gas discharge lamp and a total instrumental energy resolution of 100 meV.

The electrochemical properties of the SDC–STO bulk-heterostructure and the SDC were studied by electrochemical impedance spectra (EIS) performed with a Gamry Reference 3000 Electrochemical Workstation (Gamry Instruments, USA). The measurement was taken under open circuit voltage (OCV) mode of the cells by applying an AC voltage with amplitude of 10 mV and frequency of 0.1–10^5^ Hz on the basis of the OCV. The current density–voltage characteristics of the SDC–STO fuel cell and the SDC fuel cell were measured using an IT8511 electronic load (ITECH Electrical Co., Ltd., China) and IT7000 software was used to record the data and modulate the scan speed in the current–voltage sweep. The fuel cells were operated in the temperature range of 400–550 °C with dry hydrogen and air as fuel and oxidant (120–140 mL min^−1^), respectively.

## Results and Discussion

### Crystalline Structure and Microstructure

**Fig. 1:**
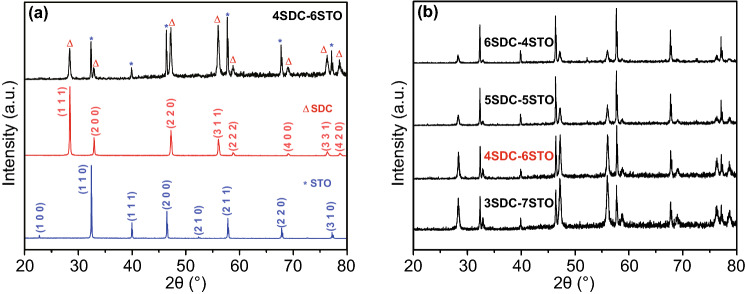
**a** XRD patterns of the prepared 4SDC–6STO bulk-heterostructure in comparison with single SDC and STO. **b** XRD patterns of four SDC–STO samples with various compositions

Figure 1a shows the XRD patterns of the prepared 4SDC–6STO (mass ratio of 4:6) in comparison with the individual SDC and STO. All these samples were well crystallized as indicated by the sharp diffraction peaks. The pattern of SDC was indexed to a cubic fluorite structure of Ce_0.8_Sm_0.2_O_1.9_ (JCPDS No. 75–0158), while that of the STO was in line with the standard cubic perovskite structure of SrTiO_3_ (JCPDS No. 37–734). As a representative sample, 4SDC–6STO demonstrated a heterostructure with combinative phase structures from SDC and STO, without any obvious peak shift or new phase. Figure 1b presents the XRD patterns of 3SDC–7STO, 5SDC–5STO, and 6SDC–4STO together with 4SDC–6STO, which shared a common feature of peak location regardless of different intensities: All diffraction peaks in each pattern can be assigned to either SDC or STO, evidencing that the two individual phases of SDC and STO coexisted in these samples without any apparent chemical interaction during the blending procedure and after the sintering process.

The grain size and distribution of the heterostructure sample were investigated by TEM. Figure S1 presents two typical TEM images of the 4SDC–6STO sample, depicting the nanoscale particles (20–50 nm) of the sample with a few small agglomerations. As shown in Fig. S1a, the 4SDC–6STO sample consisted of irregularly shaped particles with sizes from nanoscale to micrometer-scale, because of using commercial STO powders without elaborate treatment of the particle size. In the magnified region as shown in Fig. S1b, the grains of the sample showed faceted and regular shapes, with homogeneous distribution and compact contacts. A plenty of hetero-interfaces formed between the grains of SDC and STO were also observed. Figure S1c provides the selected area EDS result scanned based on Fig. S1b, confirming the elements of Sm, Ce, Sr, Ti, and O in these grains.

**Fig. 2:**
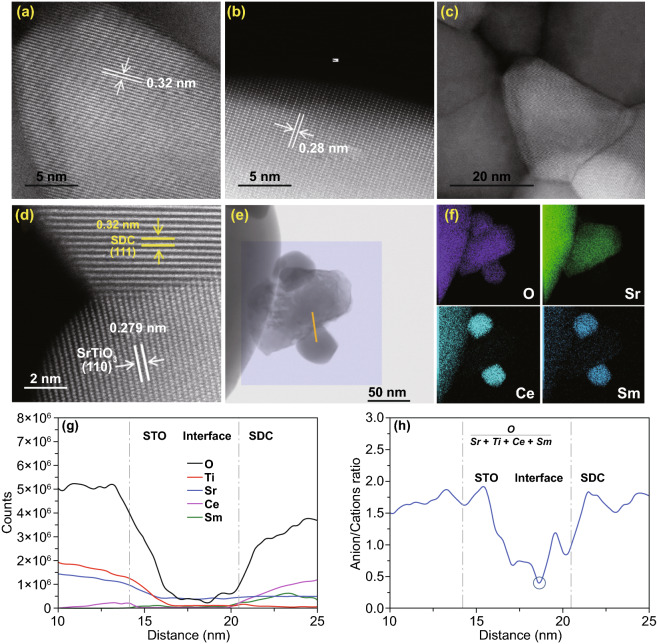
HR-TEM images of **a** SDC grains and **b** STO gains with well-defined crystalline lattice. **c** Boundaries and interfaces between the compact grains. **d** Typical well-ordered hetero-interface between SDC and STO grains. **e** Survey image for EELS mapping and positions for line scan analysis. **f** EELS mapping of the main elements O, Sr, Ce, and Sm. **g** Elemental distribution analysis and **h** the profile of atomic ratio of O/(Ce+Sm+Sr+Ti) across the interface of SDC–STO

Furthermore, the detailed microstructure of the 4SDC–6STO sample was investigated by high-resolution TEM (HR-TEM). As shown in Fig. 2a, b, the grains of SDC and STO presented well-defined crystalline lattice fringes with *d*-spacings of 0.32 and 0.28 nm, respectively. Figure 2c represents the massive boundaries and interfaces between these compacted grains. A typical interface between two grains with ordered lattice is further shown in Fig. 2d, in which the clear fringes with lattice spacings of 0.32 and 0.279 nm corresponding to the (111) plane of SDC and the (101) plane of STO can be observed, respectively. This clearly identifies the hetero-interface between the SDC and STO phases, which holds great promise to enable improved ionic conductivity of the nanocomposite via interface conduction. On the basis of the crystalline and microstructural features, the formation of a desirable bulk-heterostructure can be certified for our prepared SDC–STO samples. To study the interface characteristics of the heterostructure, STEM-EELS measurement was taken for the 4SDC–6STO sample.

Our EELS test detects two typical hetero-interfaces between SDC and STO, as shown in Fig. 2e. The elemental (O, Ti, Sr, Ce, Sm) distribution analysis and the profile of anions/cations across the hetero-interfaces of SDC–STO are attained by using line scan across the boundaries of the particles. The survey area for EELS mapping and track for line scan are marked by the rectangle and red line, respectively. Figure 2f provides the elemental mappings for O, Sr, Ce, and Sm, showing that an STO particle is sandwiched between two smaller SDC particles. In Fig. 2g, elemental distribution of Ti and Sr decrease, while those of Ce and Sm increase across the grain boundary, which indicates a hetero-interface region with ~8 nm in width between the STO and SDC particles. Figure 2h further presents the plot of atomic ratio of O/(Ce+Sm+Sr+Ti) across the interface. It is found the ratio shows obvious reduction from SDC grain to interface and from STO grain to interface, and reaches a lowest value at the distance of 18.7 nm, which is lower than that in the SDC and STO grain interior. This reflects the decreasing stoichiometry of oxygen at the hetero-interface as compared with the grain interior, signifying an improved concentration of oxygen vacancy at the SDC/STO interface region for fast ionic transport. In this way, the oxygen ion conductivity of the heterostructure can be boosted. An analogous phenomenon was also reported in a bulk-heterostructure material composed of doped ceria (Ce_0.8_Gd_0.2_O_2-δ_) and semiconductor CoFe_2_O_4_, where the depletion of oxygen vacancies was avoided at the Ce_0.8_Gd_0.2_O_2-δ_/CoFe_2_O_4_ grain boundary, leading to superior interfacial ionic conductivity [13]. This result thus reveals that ionic enhancement behavior that has been frequently detected in fluorite/perovskite planar heterostructure [9, 10] also exists in as-prepared SDC–STO bulk-heterostructure.

**Fig. 3:**
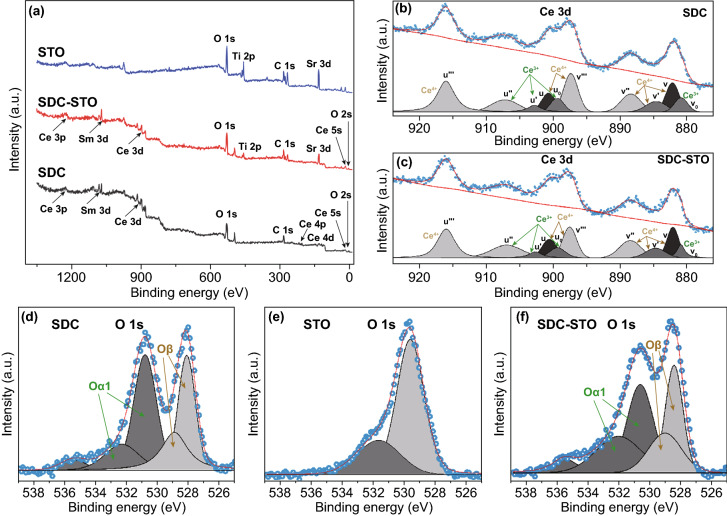
XPS results for the SDC, STO, and 4SDC–6STO samples: **a** survey spectra, **b, c** Ce 3d core-level spectra, and **d**-**f** O 1s core-level spectra

Moreover, XPS is employed to probe surface properties of the SDC, STO, and 4SDC–6STO samples. Figure 3a displays the survey spectra, in which, the 4SDC–6STO sample can be readily distinguished by the presence of Ce, Sm, O, Ti, C, and Sr characteristic peaks. To further identify the chemical states and multiple components, the Ce 3d and O 1s core-level spectra are deconvoluted by Gaussian functions and Shirley background. Figure 3b, c indicates the coexistence of Ce^4+^ and Ce^3+^ ions on the surface of SDC and 4SDC–6STO sample. The curve was deconvoluted with ten peaks, respectively. The six peaks v, v′, v′′, v′′′, u, and u′′′ were ascribed to Ce^4+^, and the remaining four peaks v_0_, u_0_, u′ and u″ were designated to Ce^3+^ [21]. It has been profoundly demonstrated that the higher concentration of Ce^3+^ suggests more oxygen vacancies were generated on the sample surface [22]. In our case, the proportion analysis of Ce at different oxidation states was calculated by integrating the corresponding peak areas, which indicates the larger Ce^3+^ content (up to 3% increase as compared to the SDC) in the 4SDC–6STO sample could contribute more oxygen vacancies on the surface.

Figure 3d, f depicts the core-level spectra of O 1s for the three samples. The peak O_α_ can be assigned to the surface oxide defects or surface oxygen species adsorbed on the oxygen vacancies, while the peak O_β_ is correlated to the lattice oxygen [23]. After calculation, it is found the relative ratio value of O_α_ and O_β_ increases from 1.13 for SDC to 1.21 for SDC–STO, revealing an increment of chemisorbed oxygen species in the heterostructure. This could lead to the ionic conduction enhancement of SDC–STO electrolyte, as the chemisorbed oxygen species are easily liberated at cell operating temperatures to expose surface oxygen vacancies for oxygen ions to transport. Combined with the EELS result, these incremented oxygen vacancies should majorly originate from the SDC/STO interface regions. According to previous illustrations [11], such enhancement of oxygen vacancies can be a result of the atomic reconstruction behavior at the SDC/STO interface between the two highly dissimilar structures (*i.e.*, fluorite and perovskite), which induces disordered oxygen area between SDC and STO. Besides, the oxygen dislocation might also occur at the SDC/STO interface because of lattice mismatch and thus possibly locally enhanced the interface oxygen vacancies.

### Electrochemical Performance

**Fig. 4:**
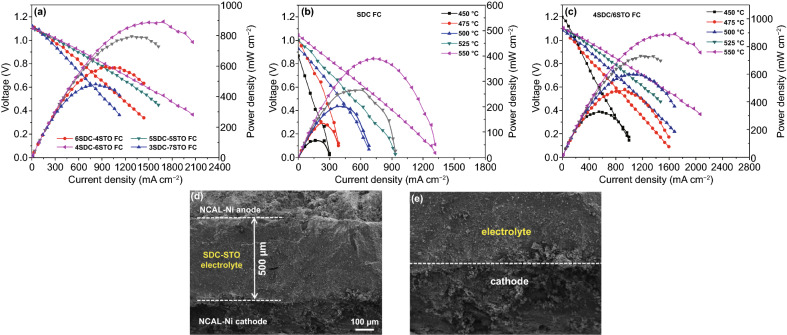
Electrochemical performance of **a** the SDC–STO SOFCs with various compositions at 550 ℃, **b** the SDC SOFC from 450 to 550 °C, and **c** the SOFC using optimal 4SDC–6STO from 450 to 550 °C. **d** Cross-sectional SEM image of the 4SDC–6STO fuel cell and **e** the electrolyte/cathode interface

The developed SDC–STO electrolytes were directly assessed in SOFCs to show the feasibility of our bulk-heterostructure approach. Fuel cell electrochemical performances were measured in the low temperature range of 450–550 °C after stabilizing the OCVs of the cells. Figure 4a presents the current–voltage (*I–V*) and current–power (*I-P*) characteristics for the SDC–STO SOFCs with various compositions at 550 °C. For comparison, the *I–V* and *I-P* curves for SDC SOFC are presented in Fig. 4b. As can be seen, all SDC–STO SOFCs exhibited superior OCV of above 1.09 V, excluding a short-circuiting concern of the cell as the semiconductor STO was applied in the electrolyte layer. The stabilized cells using the 6SDC–4STO, 5SDC–5STO, 4SDC–6STO, and 3SDC–7STO electrolytes delivered attractive peak power outputs of 595, 795, 892, and 481 mW cm^-2^ at 550 °C, respectively. Results from comparative studies revealed that: (i) the power outputs of cells are sensitive to the mass compositions of SDC and STO, and an integration of 40 wt% SDC and 60 wt% STO is proven to be the optimal composition; and (ii) the performances of SDC–STO SOFCs are apparently superior to that of the SDC electrolyte SOFC, which achieved 389 mW cm^-2^ in peak power density at 550 °C as also shown in Fig. 4b under identical operating conditions. These results can be ascribed to the effect of interfacial ionic conduction: on the one hand, the SDC–STO bulk-heterostructure created transport channels for ions at the hetero-interface region and thus gained improved ionic conductivity compared to the single SDC, resulting in higher fuel cell performance than SDC; on the other hand, various mass ratios of SDC and STO will lead to different amounts and distribution of hetero-interface in these SDC–STO samples for ionic conductivity promotion, therefore leading to strikingly diverse fuel cell power densities. In this sense, at the optimal ratio of 4:6, the grains of SDC and STO can be perfectly matched and distributed in the 4SDC–6STO sample to form more sufficient hetero-interface for better conductivity promotion as compared to other three samples.

Furthermore, the optimal 4SDC–6STO electrolyte was evaluated in fuel cell at 450, 475, 500, and 525 °C. As shown in Fig. 4c, the maximum power density of the 4SDC–6STO cell went up from 328 mWcm^-2^ at 450 °C to 892 mW cm^-2^ at 550 °C as a result of the thermally activated ion transportation in SDC–STO, accompanied by high OCVs maintaining levels above 1.09 V at each testing temperature. Compared with the state-of-the-art SOFCs with ESB-GDC (Er_0.4_Bi_1.6_O_3_-Gd_0.1_Ce_0.9_O_1.95_) thin-film electrolytes that rendered extraordinary 1–2 W cm^-2^ at 550-650 °C, our 4SDC–6STO fuel cell presents lower power densities because of the thicker ceramic electrolyte [2]. Even so, the attained favorable output of 892 mW cm^-2^ in our first demonstration still reveals the massive potential of 4SDC–6STO for LT-SOFC electrolyte uses. The stability for the SDC and 4SDC–6STO fuel cells was assessed at 500 °C for a duration of 18 h, respectively, under a same stationary current density of 100 mA cm^−2^ (Fig. S2). Both cells can be steadily demonstrated for ~14 h. Their working voltages show a similar degradation during the initial period and gradually approach a stable state. These cell performance results confirm the feasibility of SDC–STO bulk-heterostructure as an electrolyte. By integrating SDC with STO in a three-dimensional heterostructure, the SDC–STO system can be successfully demonstrated in LT-SOFCs. This erased the regret that the promising fluorite/perovskite heterostructure with high ionic conductivities failed to be used in SOFC in the past ten years. More significantly, the SDC–STO bulk-heterostructure manifested its potential to enable high fuel cell performance at low temperatures while without causing any short circuit problem. To certify the enhanced ionic conductivity and suppressed electronic conductivity in the best-performance sample, our study offers deeper insights into the electrical properties of the 4SDC–6STO below.

The cross-sectional SEM images of the 4SDC–6STO fuel cell acquired after on-line sintering and before operation are shown in Fig. 4d, e, to show the morphology of electrolyte and electrodes. As can be seen, the 4SDC–6STO electrolyte with a thickness of around 500 μm is well adhered to the porous electrodes. Due to simple assembly procedure and *in situ* sintering, the electrolyte layer is not as dense as the conventional YSZ electrolyte, but it still can support a good electrolyte functionality as proved by the fuel cell performance. To check whether there is fuel penetration into and through the electrolyte layer, the H_2_-permeation current test of the NCAL-Ni/4SDC–6STO/NCAL-Ni cell was carried out at 550 °C. The current density is extremely low as ~0.023 μA cm^-2^ as shown in Fig. S3, certifying that there is barely H_2_ penetration into and through the cell, as a solid evidence to prove that the electrolyte is gas-tight.

### Ionic Conductivity and AC Impedance Analysis

Unlike traditional electron-insulating electrolytes, the SDC–STO used in our study involved a semiconducting STO that possesses considerable electronic conductivity at 400-600 °C in reducing condition, as shown by the temperature-dependent conductivity at different oxygen partial pressures (pO_2_) in Fig. S4. This means it would be improper to use the AC impedance technique for ionic conductivity testing, as electrons from STO would have dramatically affected the measured ohmic resistance (*R*_ohm_) and grain-boundary resistance (*R*_gb_) by EIS. Therefore, in this work, the ionic conductivity of 4SDC–6STO was studied by an unconventional method from which ohmic law was used to analyze and calculate the ionic conductivity value based on the attained *I–V* polarization curves shown in Fig. 4c.

**Fig. 5:**
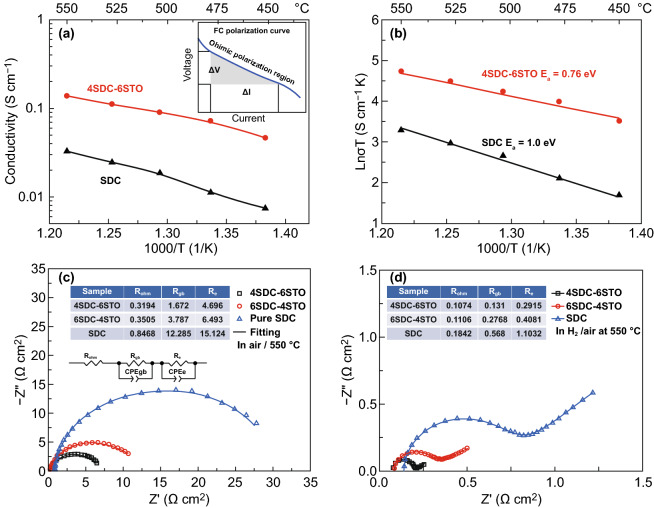
**a** Ionic conductivity and **b** activation energy plots of 4SDC–6STO and SDC as a function of 1000/T obtained from *I–V* polarization curves from 450 to 550 °C. Impedance spectra of **c** SDC, 6SDC–4STO, and 4SDC–6STO electrolyte pellets measured in air 550 °C with the equivalent circuit model for fitting. **d** Three fuel cells based on pure SDC, 6SDC–4STO, and 4SDC–6STO measured in H_2_/air 550 °C (inset: simulated parameters)

As known, the linear part of a polarization curve at low-intermediate current region reflects the total ohmic polarization loss (ΔV_ohm_) of the tested cell, which is caused by the ohmic resistances of electrolyte and electrodes [24]. In this study, the total ohmic resistance obtained from polarization curve can be approximately equal to the ionic resistance of SDC–STO electrolyte, because the electronic resistance of electrodes (NCAL/oxidized Ni-foam) is negligible in contrast to the ionic resistance of SDC–STO electrolyte. Thus, the area specific resistance (*R*_ASR_) of the 4SDC–6STO electrolyte can be expressed as *R*_ASR_=Δ*V*_ohm_/Δ*I*_ohm_ in terms of Δ*V*_ohm_ and the corresponding current drop (Δ*I*_ohm_) [25], which is actually the slope of the current–voltage characteristic curve at the ohmic polarization part (as the inset of Fig. 5a illustrates). By this, the ionic conductivity (σ_i_) of 4SDC–6STO and SDC electrolytes can be estimated according to the following equation based on the *I–V* curves:

1 $$\sigma_{i} =
								\frac{L}{{R_{{{\text{ASR}}}} \times S}} = \frac{{\Delta
								I_{{{\text{ohm}}}} \times L}}{{\Delta V_{{{\text{ohm}}}} \times
								S}}.$$

The obtained values of σ_i_ as a function of temperatures are summarized in Fig. 5a. As can be seen, pure SDC exhibited an ionic conductivity of 0.007–0.03 S cm^-1^ at 450–550 °C, which is almost consistent with previous reports [26, 27] and thus suggests the validity of the used methodology for conductivity evaluation, while 4SDC–6STO revealed ionic conductivity that was almost four times higher, 0.05–0.14 S cm^−1^ within the same temperature range. The apparent enhancement of ionic conductivity was thus observed in the materials after SDC was mixed with STO, substantiating that remarkable ionic conductivity can arise in an SDC/STO system not merely in planar but also in bulk-heterostructure. The ionic conductivity of 4SDC–6STO was superior to a series of well-known O^2-^ conducting electrolytes (SDC ~0.01 S cm^−1^ at 550 °C, ceramic YSZ 0.13 S cm^−1^ at 1000 °C, GDC/YSZ mixture film ~0.1 S cm^−1^ at 1000 °C, and thin-film YSZ 0.005 S cm^−1^ at 500 °C) [28–33]. According to previous reports with respect to YSZ/STO and our HR-TEM inspection for the hetero-interfaces, the extraordinarily high ionic conductivity is very likely a result of the increased oxygen vacancies at the grain-interface between the two dissimilar structures of fluorite and perovskite. This is different from the cases for conventional electrolytes YSZ and SDC, in which grain-boundary/interface conduction is a restraining factor and the oxygen ion transport is mainly achieved by the O^2-^ migrating via a vacancy mechanism in the cubic fluorite lattice, and unlike the YSZ/STO and SDC/STO planar heterostructures that rely on one single high-mobility plane for ionic acceleration. In addition, the high ionic conductivity may also include the contribution of protons, as proton shuttle has been reported as a crucial conduction mechanism in CeO_2_-based materials [34]. Furthermore, the activation energy (*E*_a_) for the ionic conduction of 4SDC–6STO and SDC was calculated according to Arrhenius equation *σT=A*exp[−*E*_a_/(*kT*)]. As presented in Fig. 5b, the *E*_a_ of 4SDC–6STO exhibits apparently smaller value (0.76 eV) than that of SDC (1.0 eV), which means that, in addition to enhancing the interface ionic conduction, the bulk-heterostructure could also reduce the activation energy for ionic transport. This behavior has been detected previously in YSZ/STO heterostructure [9], ascribing to the partial occupancy and high disorder in the interface oxygen plane that decreases the energy for O^2-^ migration. Compared to the result obtained by SDC, the reduced *E*_a_ of 4SDC–6STO reflects its superiority of maintaining substantial ionic conductivity for low-temperature operation. Thus, the 4SDC–6STO fuel cell can demonstrate considerable performance even at 450 °C.

In addition, EIS measurement of the 4SDC–6STO, 6SDC–4STO, and pure SDC electrolyte pellets was taken at 550 °C in air atmosphere with Ag paste on both sides, to study the influences of heterostructure on grain-boundary process, as shown in Fig. 5c. An equivalent circuit of *R*_ohm_ (*R*_gb_CPE_gb_)(*R*_e_CPE_e_) was employed to fit the experimental data, where *R* represented a resistance and CPE was the constant phase element representing a non-ideal capacitor. As summarized in the insetted table of Fig. 5c, the simulated ohmic resistance (*R*_ohm_) are around 0.32, 0.35, and 0.85 Ω cm^2^ for 4SDC–6STO, 6SDC–4STO, and pure SDC pellets, respectively, indicating distinct difference because of different amounts of electron-conducting STO in the three samples. The obtained grain-boundary resistance (*R*_gb_) of 4SDC–6STO and 6SDC–4STO (1.67 and 3.79 Ω cm^2^) are much smaller than that of SDC (12.28 Ω cm^2^), which should be majorly due to the enhanced ionic conductivity at hetero-interface/grain boundary through interfacial conduction. The EIS results suggest that the formation of heterostructure can significantly improve both bulk and grain-boundary conduction of SDC.

Moreover, EIS of the 4SDC–6STO, 6SDC–4STO, and pure SDC fuel cells were also measured in an H_2_/air fuel cell operating condition at 550 °C. As can be seen in Fig. 5d, the impedance curves of the three devices presented a same shape feature with an intersection in the high-frequency region and a semicircle located at intermediate frequencies followed by an arc fall at low frequencies, corresponding to *R*_ohm_, *R*_gb_, and the electrode polarization resistance (*R*_p_), respectively. The simulated values for *R*_ohm_, *R*_gb_, and *R*_p_ as concluded in the table of Fig. 5d are smaller than those in Fig. 5c, which should be due to the fact that in H_2_/air atmosphere proton conduction can be involved and electrode reaction is faster than that in air atmosphere. It is also found that the *R*_ohm_, *R*_gb_, and *R*_e_ values present a reducing tendency from the single SDC to the two SDC–STO samples, manifesting that the bulk-heterostructure facilitates fuel cell performances from two aspects: enhanced ionic conduction and electrode activity as compared to pure SDC. Considerable difference occurs at the value of *R*_gb_ and *R*_e_, indicative of substantially promoted grain-boundary conduction and electrode reaction. Particularly, it is found the *R*_p_ of 4SDC–6STO cell has significantly smaller values than the other two samples, as a reflection of rapid electrode reaction activity of the cell. This behavior is regarded as a beneficial consequence of the highest ionic conductivity of 4SDC–6STO that contributes to the charge transport at the electrolyte/electrode interface; in addition, the STO of the electrolyte layer can yield a mass of STO/NCAL particle contacts and form massive catalytic reaction sites for extending the electrode triple-phase boundaries, which is conducive to improving HOR and ORR kinetics of the electrodes leading to declined *R*_p_. In this sense, constructing bulk-heterostructures consisting of SDC and STO will not only achieve remarkable ionic conductivity, but also give rise to promoted electrode reaction activity rather than inducing the concerned short-circuiting issue.

### Electronic Subtraction by Metal–Semiconductor Junction

Above studies reveals that the developed SDC–STO bulk-heterostructure was successfully applied in LT-SOFC with high performance and remarkable ionic conductivity was detected with respect to 4SDC–6STO. From a conventional point of view toward electrochemistry devices, semiconductor materials possessing electronic conduction are extremely unfavorable in electrolytes for realizing normal operation of SOFCs, as the short circuit issue would cause serious OCV and power output losses when electrons transport from anode to the semiconductor phase [35, 36]. However, intriguingly, our findings certified it is feasible to demonstrate an SDC–STO electrolyte in SOFC to gain high OCVs and power densities, presenting completely different phenomena departing from the conventional theory. More crucially, the common problem of the SDC electrolyte, which always suffers from a reduction of Ce^4+^ to Ce^3+^ in H_2_ atmosphere and causes deterioration of OCV, has been eliminated in our case as indicated by the high OCVs (≥1 V) of the cells. These outcomes suggest that the concerned electron passage from the NCAL anode to the SDC–STO electrolyte was blocked by some effects during fuel cell operation.

**Fig. 6:**
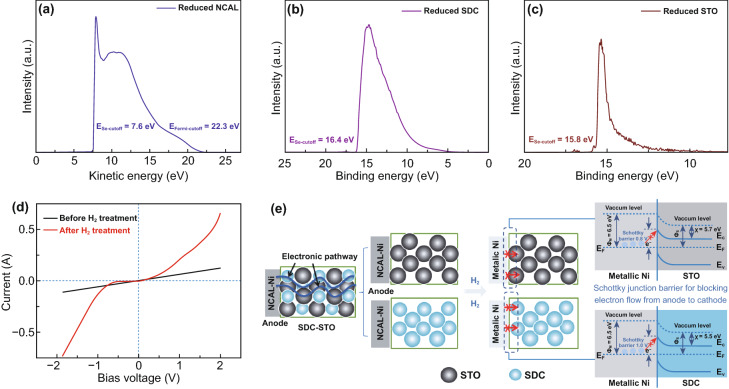
UPS plots of **a** NCAL, **b** SDC, and **c** STO pellets treated in H_2_ at 550 °C. **d** Current response as a function of bias voltage for the NCAL-Ni/4SDC–6STO half-cell tested at 550 °C before and after providing H_2._ **e** Schematic diagram of the SDC–STO fuel cell reveals the electronic blocking mechanism by Schottky junction effect at anodic NiCo/electrolyte interface

As known, the anodic NCAL can be easily reduced when hydrogen is supplied [19]. This will generate metallic Ni/Co alloy at the anode/electrolyte interface and possibly form some kind of interaction between Ni/Co and the electrolyte. Considering the semiconductor nature of STO, it can be inferred that in our fuel cell a metal–semiconductor contact (M–S contact) between anodic Ni/Co and STO was established, and this might also occur between Ni/CO and SDC. This might set up a Schottky junction involving a space charge region to prevent electrons from passing through. Generally, the M–S contact includes Schottky contact and ohmic contact, determined by the work functions for metal (Φ_m_) and the semiconductor (Φ_s_), which is the distance between the Fermi level and the vacuum level. If a semiconductor and a metal, for which Φ_m_>Φ_s_, form contact, the electrons close to the metal will leave the semiconductor until the condition of thermal equilibrium is again reached. This leads to a layer depleted of electrons and a space charge region in the semiconductor, setting up a barrier for further electron transition across the contact region, which is called Schottky junction [37]. To examine whether the Schottky junction is possible between Ni/Co alloy and the electrolyte layer in our device, the UPS spectra of the treated NCAL, STO, and SDC (treatment in H_2_ at 550 °C for 5 h before cooling down to room temperature in protective gas) were measured. The tested spectral data are presented as a function of the kinetic energy or binding energy as shown in Fig. 6a, b, c. The work function of the reduced NCAL, which is majorly metallic Ni/Co, and the treated STO and SDC were determined based on the secondary-electron cutoff and Fermi level cutoff in the spectra, giving values of 6.5, 5.4, and 4.8 eV, respectively.

The obtained work function parameters met Φ_m_>Φ_s_ for both NiCo/STO and NiCo/SDC contacts, indicative of a possibility of Schottky contacts formed in our device at the anode/electrolyte interface. To show the blocking effect of Schottky junction at the interface between the Ni/Co and SDC/STO layer, the junction barrier height was studied. As reported, the Schottky barrier height for electrons moving from the metal to semiconductor (anode to electrolyte in our cell) is determined by the difference of the Φ_m_ and the electronic affinities (χ) for semiconductors [37]. Therefore, the energy band gap (*E*_g_) of the STO and SDC samples treated by H_2_ at 550 °C was attained using UV–Vis absorption spectroscopy (Fig. S5), and the valence band (VB) maxima can be defined from the UPS results based on the second-electron and Fermi cutoffs, from which, the electronic affinities of STO and SDC were obtained as 5.7 and 5.5 eV, respectively. In this way, the barrier heights for metal/STO and metal/SDC Schottky junction are calculated as 0.8 and 1.0 eV, respectively, which can create potential barriers for blocking the electron flow from anode to cathode.

It is worth noting that the interface states located in the metal/semiconductor region may weaken the Schottky barrier height and even change the Schottky contact into ohmic contact during fuel cell operation. Therefore, for further confirming the establishment of Schottky contact in our device, the *I–V* characteristics of a NCAL-Ni/4SDC–6STO half-cell were measured at 550 °C by applying swept bias voltage (−2~2 V) to the half-cell and recording the response current. First, the *I–V* curve was tested without using H_2_ to treat the cell. Subsequently, the NCAL side was subjected to a H_2_ treatment for 30 min to form metallic Ni/Co, followed by providing protective gas N_2_ to keep the cell in a static state to measure its voltage–response current curve. As shown in Fig. 6d, the current shows a linear response with the variation of voltage before H_2_ treatment, reflecting that there is no junction effect between the NCAL anode and 4SDC–6STO in fuel cell non-operating status. After supplying H_2_ to the NCAL anode to simulate the operating status, the *I–V* curve exhibits an apparent rectification behavior, with a very small saturation current as response to the reverse bias voltage (−1~0 V) and succedent rapidly increased current under higher bias voltage >−1 V, which is in good agreement with the Schottky equation. These confirm the formation of Schottky junction between the reduced NCAL (metal phase) and 4SDC–6STO.

On basis of above study, a working mechanism of the SDC–STO fuel cell based on the Schottky junction effect is proposed. Figure 6e schematically shows the electronic subtraction process in our device and the Schottky junction at the NiCo/STO and NiCo/SDC interfaces, in which we think the Schottky barrier plays the major role in electronic suppression. Before the fuel cell operation, there were electronic channels continuously throughout the fuel cell device (indicated by the wavy lines) and such channels were classified into two electronic pathways along the anode-to-STO and along the anode-to-SDC pathways, separately. When H_2_ was provided to the anode, the elemental Ni/Co in NCAL was reduced into metallic Ni/Co and in situ formed Schottky junctions with STO and SDC. According to the attained work functions and electronic affinities, the energy band diagrams for the metal–semiconductor contacts at the anode/electrolyte interfaces are illustrated in Fig. 6e, in which the barrier heights of the NiCo/STO and NiCo/SDC junctions can prevent the electron flow from transporting across the junction [37, 38]. This would block the electronic passage from the anode to the electrolyte and effectively avoid the fuel cell short circuit risk, therefore guaranteeing high OCVs and power outputs of the cells.

## Conclusions

In summary, a series of bulk-heterostructure SDC–STO with various compositions were developed in our work for LT-SOFC electrolytes application via a facile preparation procedure. Material characterization verified the desirable bulk-heterostructure of the sample and sufficient interfacial contacts between SDC and STO with enriched oxygen vacancy concentration. When applied as the electrolytes in fuel cells, the developed SDC–STO bulk-heterostructures exhibited competent electrolyte functionality. The fuel cell with optimal sample 4SDC–6STO achieved a peak power density of 892 mW cm^-2^ along with high OCV of 1.1 V at 550 °C. In electrical studies, a remarkable ionic conductivity of 0.05–0.14 S cm−^1^ at 450 to 550 °C was detected in the sample, which is four times higher than that of pure SDC. EIS results revealed the small grain-boundary and electrode polarization resistances of 4SDC–6STO played the important role in resulting the high performance. To interpret the high OCVs and power outputs, our further investigation measured the work functions and electronic affinities of the materials to describe the electronic subtraction process in our devices based on a Schottky junction effect. The successful rollout of the fluorite/perovskite heterostructure system in this study points out a new feasible way to develop advanced electrolytes for LT-SOFCs.

## Supplementary information

Supplementary material 1 (PDF 358kb)
