# Supplementary material for: A Bulk-Heterostructure Nanocomposite Electrolyte of Ce0.8Sm0.2O2-δ–SrTiO3 for Low-Temperature Solid Oxide Fuel Cells
Source: Nanomicro Lett. 2021 Jan 4;13:46. doi: 10.1007/s40820-020-00574-3 (PMC8187505; doi:10.1007/s40820-020-00574-3)
Supplement: Supplementary file 1 — Supplementary material 1 (PDF 358kb) [file 40820_2020_574_MOESM1_ESM.pdf]

Supporting Information for

A Bulk-Heterostructure Nanocomposite Electrolyte of $Ce_{0.8}Sm_{0.2}O_{2-\delta}$-$SrTiO_3$ for Low-Temperature Solid Oxide Fuel Cells

Yixiao Cai[1, 2], Yang Chen[1], Muhammad Akbar[2], Bin Jin[2], Zhengwen Tu[2], Naveed Mushtaq[2], Baoyuan Wang[2], Xiangyang Qu[1], Chen Xia[2, *], Yizhong Huang[3, *]

[1]State Key Laboratory for Modification of Chemical Fibers and Polymer Materials, Key Laboratory of High Performance Fibers & Products, Engineering Research Center of Technical Textiles, Ministry of Education, College of Materials Science and Engineering, Donghua University, Shanghai 201620, P. R. China

[2]Key Laboratory of Ferro and Piezoelectric Materials and Devices of Hubei Province, Faculty of Physics and Electronic Science, Hubei University, Wuhan, Hubei 430062, P. R. China

[3]School of Materials Science and Engineering, Nanyang Technological University, Singapore, 639798 Singapore

*Corresponding authors. E-mail: chenxia@hubu.edu.cn (Chen Xia); yzhuang@ntu.edu.sg (Yizhong Huang)

Supplementary Figures

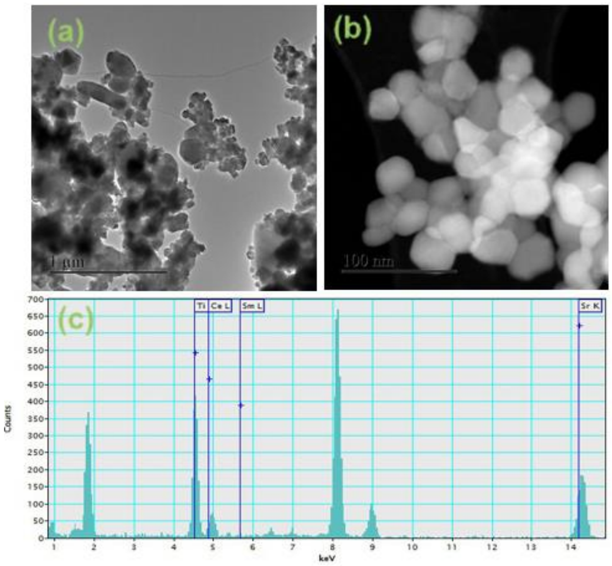

Fig. S1 TEM images of 4SDC-6STO at (**a**) low-magnification and (**b**) high-magnification; (**c**) EDS of the 4SDC-6STO sample acquired based on the high-magnification TEM

Two typical HR-TEM images and the corresponding EDS result of 4SDC-6STO, showing the grain size and distribution of the sample. The grains of the sample showed faceted and regular shapes, with uniform distribution and compact contacts. A plenty of hetero-interfaces formed between the grains of SDC and STO were also observed.

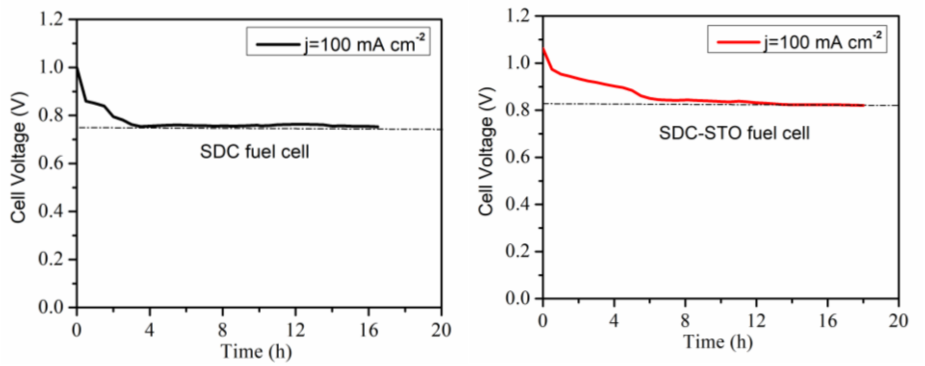

Fig. S2 Stability demonstration of SDC and 4SDC-6STO SOFCs at a fixed current density of 100 mA cm^{-2} at 500 °C

The stability demonstration of SDC- and 4SDC-6STO-based SOFCs at a fixed current density of 100 mA cm^{-2} at 500 °C for ~18 h. The working voltages for the two single cells display a degradation during the initial period and gradually approach a stable state. The SDC-based cell shows a constant working voltage of 0.75 V, while 0.84 V for 4SDC-6STO-based cell.

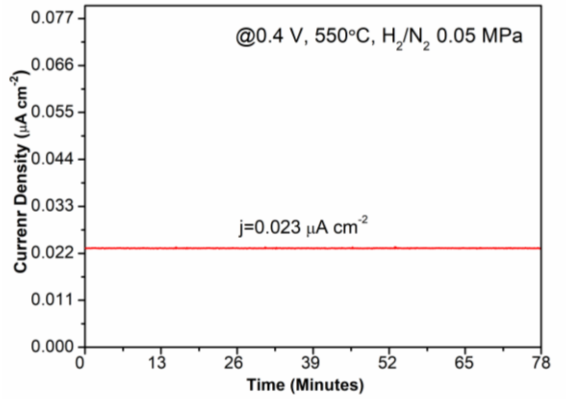

Fig. S3 H_2-permeation current test of the NCAL-Ni/4SDC-6STO/NCAL-Ni cell

The H_2-permeation current measurement was performed on an NCAL-Ni/4SDC-6STO/NCAL-Ni cell to check whether there is fuel penetration into and through the electrolyte layer. The cell was kept in the oven at 550 °C, whereafter H_2 and N_2 (both with 0.05 Mpa pressure) were provided to both surfaces of the cell with high flow rate gas flow (200 mL min^{-1}) for 2 h, until the OCV decrease to below 0.2 V. Then an external potential of 0.4 V was provided to the cell by source-meter (Keithley 2400) and the current-time curve was recorded, which can directly reflect the permeation situation of the electrolyte. The current density is extremely low as ~0.023 μA cm^{-2},

certifying there is barely penetration of H_2 into and through the cell. This authenticates that the electrolyte is gas-tight.

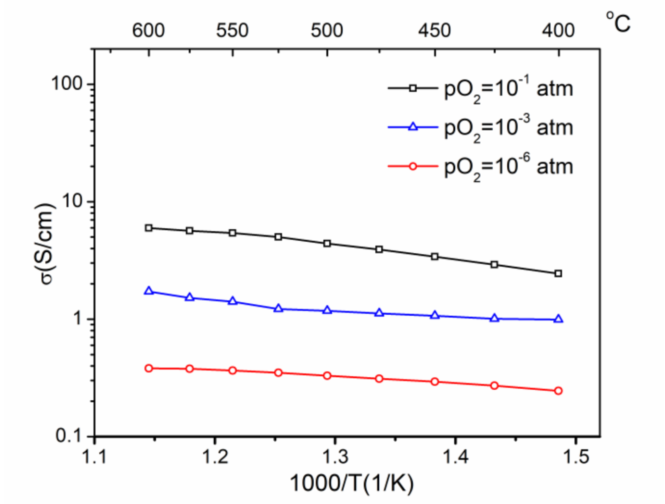

Fig. S4 Temperature dependence of the conductivity for STO sample at difference oxygen partial pressure pO_2

The temperature dependence of the electrical conductivity for the STO sample at three different oxygen partial pressure (pO_2) was measured at 400-600 °C by 4-probe DC measurement. The STO sample exhibits considerable electronic conductivity at 400-600 °C in reducing condition ($pO_2=10^{-1}$ to 10^{-6} atm).

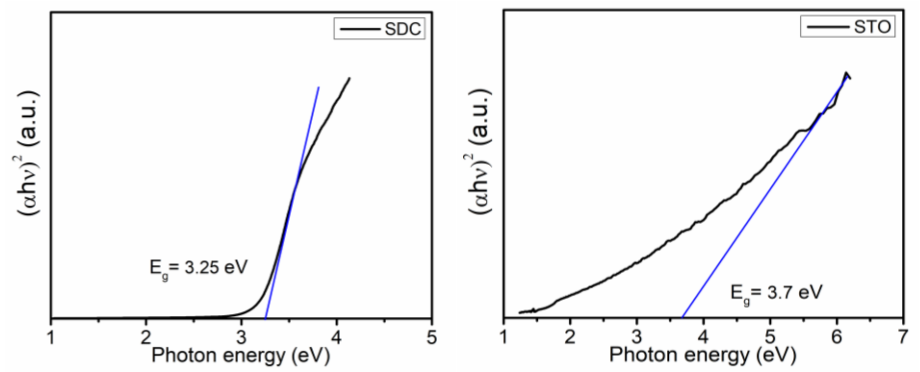

Fig. S5 Bandgap values for the SDC and STO sample treated in H_2 derived from UV-vis absorption spectra

The UV-vis absorption spectra of the SDC and STO sample treated in H_2 at 550 °C were received by a UV3600 spectrometer (MIOSTECHPTY Ltd.). Based on the results, the bandgap can be obtained by using the Kubelka-Munk function, and the bandgaps of SDC and STO are 3.25 and 3.7 eV, respectively.
